# Danger zone

**DOI:** 10.7554/eLife.69192

**Published:** 2021-05-17

**Authors:** Zohra Butt, Ian Prior

**Affiliations:** 1 Department of Molecular Physiology and Cell Signalling, Institute of Systems, Molecular and Integrative Biology, University of LiverpoolLiverpoolUnited Kingdom

**Keywords:** tumour initiation, RAS, oncogenesis, codon bias, tumor initiation, carcinogenesis, Mouse

## Abstract

What level of Ras genes activity leads to the development of cancer?

**Related research article** Li S, Counter CM. 2021. Signaling levels mold the RAS mutation tropism of urethane. *eLife* **10**:e67172. doi: 10.7554/eLife.67172

When a cell multiplies, differentiates or dies, it relies on a number of complex signalling networks. In turn, mutations in nodes that increase or decrease communication through these networks frequently result in diseases. An example is the Ras gene family, which is often mutated in cancer: activating mutations at certain Ras codons leads to cells proliferating and forming tumours (Prior et al., 2020). However, too much activation can trigger safety mechanisms and cause the cell to die. How much Ras activity is enough to drive cancer is therefore a fundamental question.

For a long time it was assumed that any mutation that activated Ras proteins would lead to disease. New evidence, however, has revealed that local cellular and disease context creates important differences between Ras mutants (Killoran and Smith, 2019; Haigis et al., 2019). In one study in mice, for example, out of twelve different mutations introduced in equal quantities in a Ras gene called *KRAS*, only five led to the animals developing lung tumours (Winters et al., 2017). Intriguingly, which mutation drives disease was different depending on the type of cancer, and the genetic background of the mouse strain. These data imply mutation-specific differences in Ras biology.

Now, in eLife, Siqi Li and Christopher Counter from Duke University report having described the optimal conditions in which various Ras mutations operate (Li et al., 2018). According to a previously proposed ‘sweet spot’ model, there is a level of Ras activity high enough to promote tumour formation, but not to lead to cell death (Li and Counter, 2021). To examine this further, a classic mouse cancer model was exposed to urethane, a chemical found in fermented foods that consistently generates a codon Q61* mutation in *KRAS* and leads to Ras-driven lung cancer (Westcott et al., 2015; Dwyer-Nield et al., 2010). Codon Q61* mutations are known to lead to more Ras activity than codon G12* mutations (Burd et al., 2014; Figure 1); this suggests that codon Q61* mutations, rather than G12*, have optimal levels of Ras signalling in this urethane-induced cancer model.

**Figure 1.**
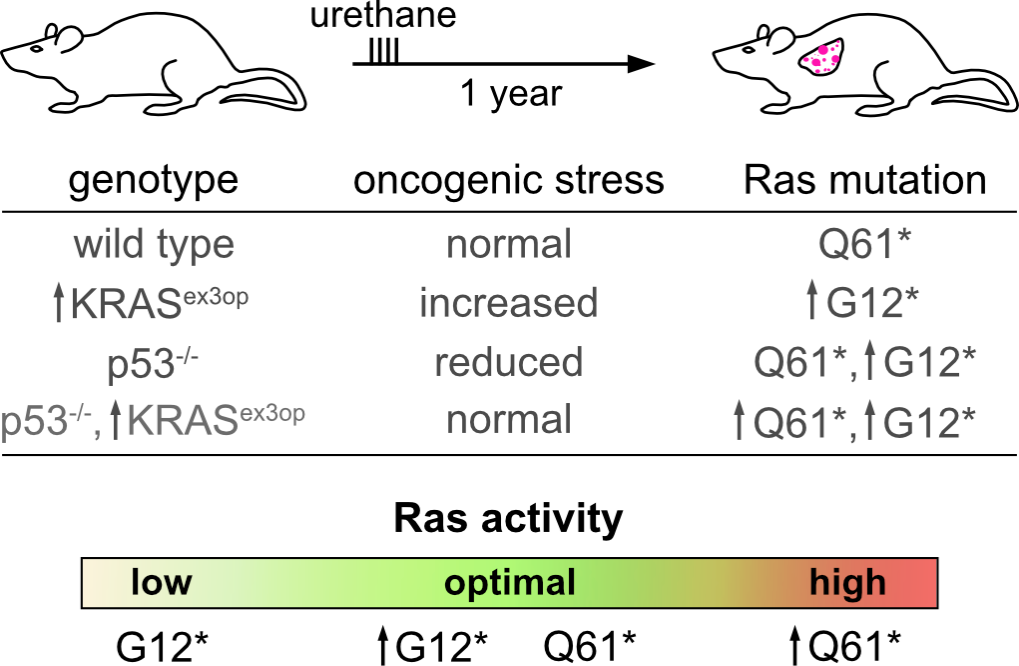
Optimal Ras signalling is required for tumour development. Li and Counter investigated the impact of Q61* and G12* mutations in the Ras gene *KRAS* on wild-type and mutant mice. Q61* and G12* mutations respectively lead to a large and moderate increase in the activity of the gene. Wild-type mice exposed to urethane (which causes Q61* mutations) develop lung cancer after a year (top; first line of table). KRAS^ex3op^ mutant mice have raised Ras activity, and therefore increased oncogenic stress; in these animals, the G12* mutation is the main driver of tumours, because it is less active than Q61* (second line of table). Conversely, p53^-/-^ mice have decreased oncogenic stress and are able to tolerate high levels of Ras activity driven by Q61* mutations, leading to tumour growth; however, they also showed increased levels of G12* *KRAS* mutant messenger RNA (third line). p53^-/-^, KRAS^ex3op^ mutants have normal levels of oncogenic stress, and in these animals both Q61* and G12* mutation can lead to disease (fourth line). Overall, depending on the genetic background of the animal, which mutations lead to the level of Ras activity that triggers cancer varies (bottom). ↑ indicate genotypes or post-transcriptional mechanisms that increase Ras abundance.

To test if weaker G12* mutations could also induce cancer in this model, a mouse strain with increased *KRAS* expression (called KRAS^ex3op^) was exposed to urethane, artificially boosting the amount of active Ras. Even though Q61* mutations were still generated, G12* mutations were found to drive the development of tumours in these animals; this demonstrated that the switch was due to Ras biological properties, a result consistent with the sweet spot model.

Whether strong Ras signalling – which would normally induce cell death – could be moved into the optimal activity zone was explored by deleting p53 in wild type mice. This gene instructs cells to die when oncogenic stress induces unrepairable DNA damage. As predicted, p53^-/-^, KRAS^ex3op^mouse strains with increased *KRAS* expression and depleted p53 could tolerate high levels of Q61* mutations (Figure 1). Intriguingly, p53^-/-^ mice also showed an endogenous amplification of *KRAS*, which moved G12* mutations up into the optimal Ras signalling zone. This was not due to additional copies of Ras genes, but to an increase in the production of messenger RNAs carrying the G12* change.

Together, these data reveal a narrow window of cancer-causing Ras activity; this suggests that the role of specific Ras mutations, and how they are combined, needs to be considered for research design and treatment options. However, further studies ought to formally quantify how a range of Ras mutations and combinations differ in their relative activity. This will help to confirm whether the model holds true across a broader range of cancer contexts, and to more precisely determine optimal Ras activity.

The model is based on observed endpoints, after tumours have grown. Yet, it is reasonable to assume that the optimal level of Ras signalling changes as the cancer develops: for instance, Ras alleles are amplified and lost over the life history of cancer, and in response to therapy (Burgess et al., 2017). An exciting observation was the increase in *KRAS* messenger RNA to help modulate Ras activity; however, this still needs to be validated by measuring Ras protein levels. Finally, how variable levels of Ras activity then variously impact wider cancer signalling networks is a big question that remains unanswered.
